# Gut Microbiota Interventions for the Management of Obesity: A Literature Review

**DOI:** 10.7759/cureus.29317

**Published:** 2022-09-19

**Authors:** Vikram Jeet Singh Gill, Suha Soni, Manasi Shringarpure, Anusheel ., Sushant Bhardwaj, Narendra Kumar Yadav, Ankit Patel, Avaniben Patel

**Affiliations:** 1 Internal Medicine, Baylor College of Medicine, Houston, USA; 2 Medicine, Punjab Institute of Medical Sciences, Jalandhar, IND; 3 Public Health, University of Texas Health Science Center at Houston, Houston, USA; 4 Medicine, The Institute of Liver and Biliary Sciences (ILBS), Delhi, IND; 5 General Practice, Shanti Gopal Hospital, Ghaziabad, IND; 6 Medicine, Kathmandu University School of Medical Sciences, Kathmandu, NPL; 7 Medicine, Nepalgunj Medical College, Kathmandu, NPL; 8 Internal Medicine, Spartan Health Science University School of Medicine, Vieux Fort, LCA

**Keywords:** gut microbiome, probiotics and microbiome, microbiota, intestinal microbiota, dysbiosis, scfa, obesity, metabolism, gut microbiota, gut

## Abstract

The gut microbiota (GM) has been recognized as an important factor in the development of metabolic diseases such as obesity; it has been reported that the composition of the GM differs in obese and lean subjects, suggesting that microbiota dysbiosis can contribute to changes in body weight. Dysbiosis occurs due to an imbalance in the composition of gut bacteria, changes in the metabolic process, or changes in the distribution of microbiota within the gut. Dysbiosis can change the functioning of the intestinal barrier and the gut-associated lymphoid tissues (GALT). Microbial manipulation may help with preventing or treating weight gain and associated comorbidities. Approaches to this may range from dietary manipulation, which is suitable to treat the individual’s microflora, to probiotics, prebiotics, synbiotics, and fecal microbiota transplant (FMT).

## Introduction and background

Obesity is a worldwide public health problem that continues to rise rapidly and accounts for over 60% of deaths related to high body mass index (BMI) [1]. Obesity is considered a complex and multifactorial condition [2]. The association and causative role played by gut bacteria in obesity represent one of the most important findings in the field [3]. The gut microbiota (GM) is intertwined with host physiology and pathophysiology. GM has recently been recognized as an important factor in the development of metabolic diseases [4]. Changes in the composition of GM may result in a change in the relationship between the bacteria and the host, which can lead to an inflammatory process and metabolic disorders seen in obesity [5]. Initial research generally examined the microbiota composition and its relation to disease presentation, but there has recently been a shift toward the understanding of the mechanisms by which variation of the microbiota can lead to disease manifestations [6]. Our understanding of the interrelationships between GM and the development of obesity remains descriptive, and large gaps between clinical and experimental knowledge still persist. This review presents a brief introduction to GM and its mechanism of action in relation to obesity, influential factors on microbiota including dysbiosis, and interventions indicated for obesity with respect to GM.

## Review

Mechanisms of action of gut microbiota

**Figure 1:**
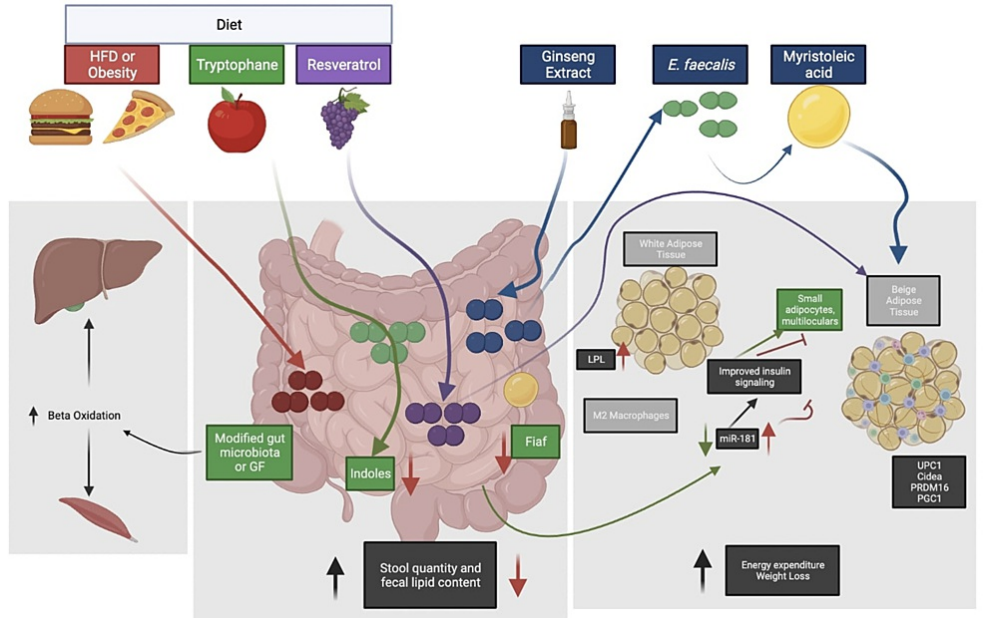
Changes in intestinal microbiota due to different triggers ultimately affecting weight storage and metabolic health in mice Adapted from [16] HFD: high-fat diet; LPL: lipoprotein lipase

GM has been shown to regulate energy homeostasis, inflammation, glucose, and lipid metabolism in various studies [7]. However, there are certain microbes in the gut whose role is still unknown [8]. Various studies have reported that GM translocates from the gut to tissues in obese type 2 diabetes mellitus (T2DM) patients, causing increased inflammation [9]. It was observed that a proper symbiotic relationship with GM reduces the incidences of increased intestinal permeability, thereby reducing chronic inflammation and increasing insulin sensitivity [10]. In a study on leptin-resistant mice, the mechanism of action of *Saccharomyces boulardii* (*S. boulardii*; yeast) was examined by daily administration of oral gavage of *S. boulardii* for four weeks. This microbe was shown to act via the putative gut-to-liver axis as well as by improving gut barrier function. These mechanisms are used by *S. boulardii *to reduce fat mass, hepatic steatosis, and systemic and hepatic inflammation in obesity and T2DM [11]. In another study on mice, it was seen that metabolites derived from tryptophan from GM controlled mir-181 expression in white adipose tissue (WAT) that in turn regulates inflammation and metabolism [12]. The study on db/db mice administered with resveratrol (RSV), a polyphenol compound that is found in grape seeds and skin, found that it had improved glucose homeostasis that was due to brown adipose tissue (BAT) and WAT browning. This process was further shown to be mediated by GM by the BA-TGR5/UCP1 pathway [13]. There is increasing evidence revealing GM's relation with energy metabolism. Studies have suggested that GM is involved in preventing obesity by enhancing the uncoupling protein 1 (UCP-1)-dependent thermogenesis [14,15]. GM mediates the action of curcumin using the UCP-1 pathway to prevent and treat obesity [16]. Intestinal microbiota (IM) compositional changes have been seen in obesity, T2DM, dyslipidemia, and non-alcoholic fatty liver disease through various studies as shown in Figure 1 [17-19]. 

IM has been shown in various studies to produce enzymes that are responsible for breaking down indigestible carbohydrates [20,21]. A recent study has shown that IM browns WAT, thereby regulating body weight and energy expenditure increase and managing insulin resistance [22,23].

Benefits and harms of gut microbiota interventions/modulation

Due to the rapidly increasing awareness about the benefits of probiotics, their current annual market growth of about 7% is expected to grow to a whopping USD 65 billion by 2024 [24]. Probiotic species such as Lactobacillus and Bifidobacterium are the safe microorganisms to use; others such as Streptococcus, Enterococcus, Bacillus, and other spore-forming bacteria are used in probiotics despite their known deleterious effects on human health [25]. There have been concerns about the long-term uses of probiotics and protein-fortified foods despite the abundant benefits of probiotics [26]. Bacterial translocation is the most dreaded issue associated with probiotics according to scientists, causing serious effects such as bacteremia, sepsis, and endocarditis [27,28]. It thus becomes extremely important to study the translocation ability before administering probiotics based on studies in vitro or in animals [29]. These effects are not very marked in healthy individuals due to the killing of bacteria by mesenteric lymph nodes; however, this mechanism is defective or absent in immunocompromised individuals, rendering translocation detrimental in such patients.

Cannon et al. conducted a study on around 200 patients over the span of 53 years; it was noted in vitro studies that monotherapy of antibiotics was sufficient for probiotic infections but the sensitivity towards vancomycin, cefazolin, and ciprofloxacin was reduced especially in Lactobacillus spp. infections [30]. Another set of patients at risk of Lactobacillus bacteremia are those with ulcerative colitis due to the loss of integrity of the mucosal barrier in the intestines [31,32]. There is an evident lack of data, and hence further studies along with an understanding of bloodstream portals on Lactobacillusinfection in immunocompromised patients are warranted urgently [33].

Gut microbiome and dysbiosis procedures

GM consists of beneficial microbes to opportunistic pathogens. Commensal bacteria colonize the intestine immediately after birth. A healthy adult gastrointestinal tract (GIT) harbors approximately 1,000 bacterial species. Firmicutes, Bacteroidaceae, Lachnospiraceae, Actinobacteria, Prevotellaceae, and Ruminococcaceae are the dominant groups of bacterial species [34]. The microbial species of the gut help in various aspects such as vitamin synthesis, digestion of large molecules, and other aspects of metabolism. However, the composition of bacterial species varies with lifestyle changes, diet modifications, and medication use [35]. Dysbiosis occurs due to an imbalance in the composition of gut bacteria, changes in the metabolic process, or changes in the distribution of microbiota within the gut. This disruption can occur in three ways as shown in Table 1.

**Table 1:** Different ways of changes in the distribution of microbiota in the gut Adapted from [36]

S. no.	Way of disruption
1	Loss of beneficial bacteria
2	Loss of overall distribution and diversity of gut microbiota
3.	Overgrowth of pathogenic bacteria

Dysbiosis can change the functioning of the intestinal barrier and the gut-associated lymphoid tissues (GALT) by allowing the passage of structural components of bacteria, such as lipopolysaccharides (LPS), which activate inflammatory pathways that may contribute to the development of insulin resistance [37]. To understand the process of dysbiosis, it is vital to first know the composition of GM in the human body. Multiple studies have shown that normal human GM mainly consists of over 1000 species, most of which belonging to classes of Firmicutes, Bacteroides, Proteus, Fusobacteria, Actinomycetes, and Verrucomicrobia [38,39]. Out of these, Bacteroides and Firmicutes predominate the flora [40]. 

The most important functions of normal healthy GM include producing short-chain fatty acids (SCFAs), producing vitamins and essential amino acids, and biodegrading of polysaccharides. A healthy gut flora maintains equilibrium and homeostasis between commensal and pathogenic bacteria and is able to return to a healthy state after modulation, like after the use of antibiotics [41]. That being said, several genetic sequencing studies and diet-induced mouse model studies suggest that an increase in the ratio of Firmicutes/Bacteroides at the phylum level is crucial for GM in obesity [39,42]. An increased ratio of Firmicutes/Bacteroids was also noted in studies that observed overweight and obese volunteers [43]. Regulation of GM occurs by energy absorption, storage of fat, regulation of circadian rhythm, chronic inflammation, etc. [44].

Short-chain fatty acids and dysbiosis

SCFAs are carboxylic acids with aliphatic tails of one to six carbons that are produced by the anaerobic fermentation of dietary fibers in the intestine by GM [45,46]. The production of SCFA plays one of the major roles relating to healthy gut bacteria. It is noted that in the intestine, a decrease in SCFA levels, due to an increase in SCFA absorption and altered healthy microbiota, is observed in obese individuals. SCFAs inhibit fat accumulation in adipose tissue, thus decreasing the levels contributing to obesity. The amount of SCFA produced in the gut rather than the composition of GM plays an important role in obesity [47]. Several intra- and inter-individual variances in GM composition make the definition of healthy microbiota complex. Table 2 shows the classification of dysbiosis into different forms [48-52].

**Table 2:** Different forms of dysbiosis of the gut along with their respective causes Adapted from [48,49,50,51,52]

Type of dysbiosis	Cause
Deficiency dysbiosis	Reduction in the beneficial bacteria such as Lactobacilli or Bifidobacteria due to an unhealthy diet or antibiotic use and can also be associated with food intolerance
Putrefactive dysbiosis	Increase in putrefactive bacteria like Bacteroides, generally resulting from rich fat and a poor fiber diet
Fermentative dysbiosis	Reduced gastric production with increased bacterial fermentative activity
Susceptibility dysbiosis	Loss of tolerance of intestinal microbiota and alterations of gut microbiota ecosystems due to a reduced amount of probiotic bacteria, increased pathogenic microbes or pathobionts, and altered motility of the intestine
Fungal dysbiosis	Overgrowth of Candida or other fungal species in the microbiota due to a diet rich in sugar and low in fiber

Gut microbiome interventions in obesity

Microbial manipulation may be employed to prevent or treat weight gain and associated comorbidities. Approaches to this include use of probiotics, prebiotics, synbiotics, fecal microbiota transplant (FMT), and other interventions. The success of these therapies largely depends on factors such as the nature of resident microbiota composition and structure and understanding of the dynamic alterations that occur over time [53]. 

Prebiotics 

Prebiotics have been studied widely for their use in treating obesity. Numerous clinical studies have shown the benefits of using prebiotics in obesity by improving appetite control and reduction of body fat [54-57]. Prebiotics are a class of nutritional compounds categorized together, not necessarily by structural affinity, but by the potential to promote the growth and/or activity of specific beneficial bacteria (probiotics) in GM [58]. In 2004, prebiotics was upgraded to include four criteria as shown in Table 3 [59].

**Table 3:** Criteria needed to be satisfied in order to be categorized as a prebiotic Adapted from [59]

S. no.	Criteria
1	Resistance to hydrolysis by mammalian enzymes, gastric acidity, and gastrointestinal absorption
2	Fermentation only by gut microbiota
3	Induce systemic or luminal effects that are beneficial to host health
4	Selectively stimulate the growth and activity of gut microbiota associated with health and well-being

Fecal Microbiota Transplantation (FMT)

FMT refers to altering the host’s gut microbiome in order to provide a therapeutic effect [60]. It involves the introduction of microbiota from a healthy donor's feces to the morbid individual’s GIT and has been used in metabolic syndrome (MS) and diabetes [61]. Various methods of FMT are employed, such as orally by upper gastric sections (UGI route) and oral capsules, nasally, and rectally by colonoscopy (LGI route) [62,63]. FMT is a radical procedure that has been successful in the treatment of patients with recurrent Clostridium difficile (C. difficile) infections, the first instance of which was documented in 1983 [64]. It is currently the treatment of choice for C. difficile diarrhea that is unresponsive to antibiotic therapy [65]. Various studies have shown that obesity is linked to a decline in microbial diversity, which in turn leads to metabolic dysfunction, and given this, FMT can be a good option to restore the diversity, which in turn may be used to treat obesity [66].

Like any other organ transplant, risks associated with transplant and donor selection are to be considered. A study in which lean donors were given oral capsules for FMT was conducted and it was found that weekly administration of FMT capsules resulted in microbiota engraftment in obese adults for 12 weeks, but no clinically significant metabolic effects were seen during this study [67]. A study on obese adults showed similar results: no significant changes in a 12-week period in both FMT- and placebo-administered groups. It was shown that FMT capsules might be safe for administration but had no effect on BMI [68]. Other studies warn of the adverse effects (AEs) of microbiota transplantation such as cytomegalovirus infection, norovirus infection, and Escherichia coli bacteremia [69]. The UGI route is known to cause complications such as nausea, vomiting, nasal congestion, and asphyxia, while the LGI route is known to cause abdominal pain, anorectal discomfort, and rectal abscess [70-74].

Bacterial Consortium Therapy

An alternative to FMT would be a well-defined microbiota that is rebalanced, comprising gut bacteria, or bacterial consortium therapy (BCT) [75]. BCT involves the use of defined drug compositions produced from clonally isolated bacteria that can trigger targeted immune responses. Specific intestinal ecosystem modulation could be performed with BCT. A recent study showed complete recovery and effects comparable to those of FMT with BCT as a substitute [76]. Bacterial consortiums are defined accurately and can be prepared based on different levels or types of dysbiosis. Patient safety in this regard is improved as the bacterial combination can be controlled for pathogenic microbes. In this context, BCT could be a safer alternative to FMT to modulate intestinal dysbiosis [77].

Phage Therapy

Bacteria-specific viruses (phages) have a great influence on the bacterial population of microbes. They have good therapeutic potential and can be used as an alternative to antibiotics or to modulate the composition of the gut flora [78]. Given the presence of our microbial ecosystem, the risks of phage therapy do not appear to be high. Phage suspensions can be prepared for both local (introduced directly in the gut) and systemic therapy, keeping in mind the amplification of phages after administration [79]. The kinetics of amplification usually depends on the concentration of susceptible bacteria, the immune responses of the host, etc. Due to these variables, the dosing and timing of administration of phage therapy have been problematic. Further studies and essential data are required and are needed to be addressed for the approval of phages for the management of obesity by the FDA [80].

Other Micronutrients

Zinc (Zn) is known to be essential for all forms of life. A group II-B metal, it is known to be involved in the functioning of more than 300 enzymes. Its deficiency has been associated with obesity, T2DM, hypertension, and coronary heart disease [81]. A study was conducted on obese rats regarding the antioxidant and metabolic effects of Zn along with branched-chain amino acids (BCAA) supplementation. Over a span of 19 weeks, male Wistar rats were fed a high-fat/fructose diet (HFD) and a standard diet (SD). It was seen that HFD-fed animals had elevated leptin, triglycerides, plasma insulin, increased weight, and abdominal fat pad than the SD-fed animal group. Surprisingly, these parameters were all reduced by Zn supplementation. It clearly demonstrated the role of Zn in metabolic dysfunction and obesity [82]. The most recent advances in the role of Zn in health and disease from 2010 to 2020 showed adipotrophic effects by the role of Zn finger proteins, Zn transporters, and Zn-alpha2-glycoprotein. This in turn depicted its role in obesity and T2DM pathogenesis [83]. Zn is directly involved in insulin secretion, modulating long-chain polyunsaturated fatty acids (PUFA), and indirectly involved in lipid metabolism in some rat studies; Zn has been shown to aid glucose uptake and inhibit free fatty acid release. Thus, it has a huge role in metabolic syndrome and obesity [84].

Retinoic acid as a metabolite of vitamin A is involved in developing visual systems and epithelial tissue. Along with its role in normal metabolism and immunity, it has recently been proven to be effective in metabolic diseases [85]. There have been multiple observational and epidemiological studies showing that obesity is related to low levels of carotenoids in circulation [86,87]. It has been demonstrated in various studies that BMI and the level of carotenoids (except lycopene) are inversely correlated [88].

Vitamin D has been known to be essential in calcium homeostasis for a healthy skeletal system [89]. Vitamin D receptor (VDR) has been highly expressed in adiposities and also responds to fat-soluble 1,25(OH)2D. A population-based study conducted in Norway showed an inverse relationship between low serum 25(OH)D levels and increased BMI [90]. In various other studies, increased body fat and higher BMI are shown to be highly related to 25(OH)D levels [91-94]. Various meta-analyses have shown an inverse relationship between body weight and vitamin D levels; however, this relationship remains largely confusing due to confounding studies warranting more in-depth research studies to definitively prove the association [91]. Most studies show that supplementation with vitamin D does not have an effect on body weight or BMI but does affect body fat distribution [92,93]. In a study on Japanese adolescents, it was shown that low serum vitamin D levels are associated with obesity and atherogenesis in adolescent boys only and not adolescent girls; hence future studies are warranted to establish a concrete relationship [92].

Folate or vitamin B9 refers to water-soluble compounds that are necessary for human health and development [95]. In a study conducted on mice by Zhao et al., chronic folate-deficient diet (CFD) induced obesity, hepatic lipid gene regulation disturbance, hypertriglyceridemia [elevated serum triglyceride (p<0.001), elevated VLDL and cholesterol levels (p<0.001)] and insulin resistance [96]. It has been shown in studies that folate and vitamin B12 supplementation is closely linked to decreased risk of stroke and cardiovascular diseases, which are sequelae of metabolic syndrome and obesity, in both men and women in the United States [97,98].

Limitations

The present literature review has a few limitations that should be taken into consideration. The study was restricted in scope in terms of language (English language studies only). We may have missed studies published in other languages that may assess the topic in different cultures and population groups. No studies published before the year 2000 have been used in writing this review. However, all types of research designs were included in this literature review.

## Conclusions

There is a large amount of heterogeneity in the data available on the subject, and the conclusion that can be drawn from the literature review is that dysbiosis can change the functioning of the intestinal barrier. Microbial manipulation may be employed to prevent or treat weight gain and associated comorbidities. Understanding the changes occurring in the GM of obese individuals and the physiological consequences of these changes is a necessary step in creating future modulation strategies and is a potential area for further research.
